# Spirofused tetrahydroisoquinoline-oxindole hybrids as a novel class of fast acting antimalarial agents with multiple modes of action

**DOI:** 10.1038/s41598-020-74824-0

**Published:** 2020-10-21

**Authors:** Noella M. Efange, Maloba M. M. Lobe, Rodrigue Keumoe, Lawrence Ayong, Simon M. N. Efange

**Affiliations:** 1 Department of Biochemistry and Molecular Biology, University of Buea, P.O. Box 63, Buea, Cameroon; 2 Centre Pasteur du Cameroon, Yaoundé, Cameroon; 3 Department of Chemistry, University of Buea, P.O. Box 63, Buea, Cameroon

**Keywords:** Biochemistry, Drug discovery, Diseases, Chemistry

## Abstract

Molecular hybridization of privileged scaffolds may generate novel antiplasmodial chemotypes that display superior biological activity and delay drug resistance. In the present study, we describe the in vitro activities and mode of action of 3′,4′-dihydro-2′H-spiro[indoline-3,1′-isoquinolin]-2-ones, a novel class of spirofused tetrahydroisoquinoline–oxindole hybrids, as novel antimalarial agents. Whole cell phenotypic screening of these compounds identified (14b), subsequently named (±)-moxiquindole, as the most potent compound in the current series with equipotent antiplasmodial activity against both chloroquine sensitive and multidrug resistant parasite strains with good selectivity. The compound was active against all asexual stages of the parasite including inhibition of merozoite egress. Additionally, (±)-moxiquindole exhibited significant inhibitory effects on hemoglobin degradation, and disrupted vacuolar lipid dynamics. Taken together, our data confirm the antiplasmodial activity of (±)-moxiquindole, and identify 3′4′-dihydro-2′H-spiro[indoline-3,1′-isoquinolin]-2-ones as a novel class of antimalarial agents with multiple modes of action.

## Introduction

Malaria is a devastating disease that causes considerable morbidity and mortality worldwide and constitutes a major public health problem in many countries. According to the World Health Organization (WHO) World Malaria Report, an estimated 228 million cases of malaria and 405 thousand deaths occurred worldwide in 2018^1^. While these figures reveal a 9% decrease in malaria burden compared to the situation in 2010, it is obvious that the disease remains a major public health threat, especially in Africa which accounts for 93% of the cases. As recommended by the WHO, artemisinin combination therapies (ACTs) are currently the frontline treatment for malaria^2,3^. Unfortunately, the emergence and rapid spread of artemisinin resistance threatens the efficacy of ACTs^4–6^ and highlights the need for newer antimalarials, preferably with novel mechanisms of action, to reinforce the antimalarial drug armamentarium.

**Figure 1:**
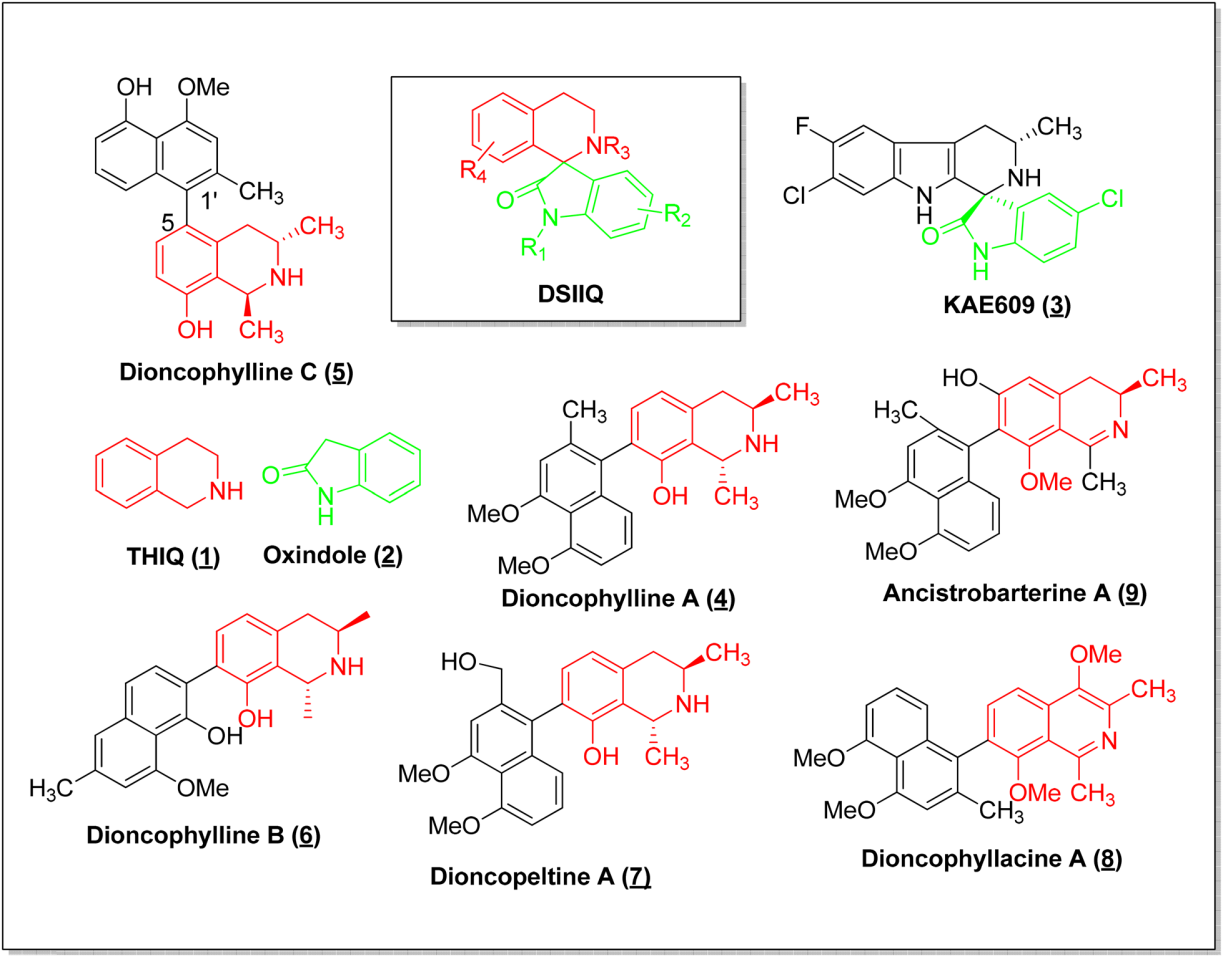
NITD 609 (Spiroindolone), naphthylisoquinolines and the hybrid scaffold.

Antimalarial agents that attack multiple biological targets have the potential to display superior pharmacological profiles and delay the emergence of drug resistance. One approach to the design of compounds with pleiotropic effects is by the molecular hybridization of privileged scaffolds. A privileged scaffold as defined by Evans et al.^7^ is a structural subunit common to numerous biologically active compounds and that has high affinity against a diverse set of targets. Consequently, privileged scaffolds have become important starting points in drug discovery efforts; moreover, the combination of two or more of these scaffolds, termed molecular hybridization, has been used to design multi-target agents for a number of applications including malaria^8,9^. In our search for novel antimalarial scaffolds, we therefore chose to study the recently described 3′,4′-dihydro-2′H-spiro[indoline-3,1′-isoquinolin]-2-ones (DSIIQs) which had been designed as molecular hybrids of two privileged scaffolds, tetrahydroisoquinoline (THIQ) and oxindole (OX). These molecular hybrids offered a particularly attractive target for investigation because they combine privileged scaffolds that occur separately in mechanistically disparate groups of antimalarial agents, spiroindolones and naphthylisoquinolines, as revealed below:

1. DSIIQs are structurally similar to the spiroindolones, otherwise known as THβC-substituted oxindoles (exemplified by KAE609, compound 3 in Fig. 1), a promising new class of antimalarials with a novel mechanism of action. Compounds in this class, represented by KAE609 (cipagarmin) which is currently in clinical trials, inhibit *Pf* ATPase-4 and have been shown to inhibit all erythrocytic stages of the *P. falciparum* life cycle^10,11^. Structurally, the spiroindolones may be regarded as THβC-substituted oxindoles while the DSIIQs may be viewed as THIQ-substituted oxindoles.
2. Similarly, DSIIQs are structurally related to the naphthylisoquinolines (NIQs) (exemplified by compounds 5–9 in Fig. 1), a class of structurally diverse plant secondary metabolites characterized by the presence of a THIQ scaffold substituted with a naphthyl group. The NIQs display remarkable anti-malarial, anti-trypanosomal, anti-leishmanial, fungicidal, molluscicidal, larvicidal, insecticidal, spasmolytic, and anti-HIV activities^12^. The NIQs display potent activity against the asexual erythrocytic stages of *P. falciparum* and *P. berghei *in vitro^13,14^; are active against the liver stages of *P. berghei*^15^ and are curative in vivo^16^. Moreover, the antimalarial activity appears to reside in the THIQ fragment and the mode of action of the NIQs appears to be primarily inhibition of hemoglobin degradation^17^.

The present study describes the antimalarial activity of these recently described hybrids and their modes of action.

## Materials and methods

### Test compounds

**Figure 2:**
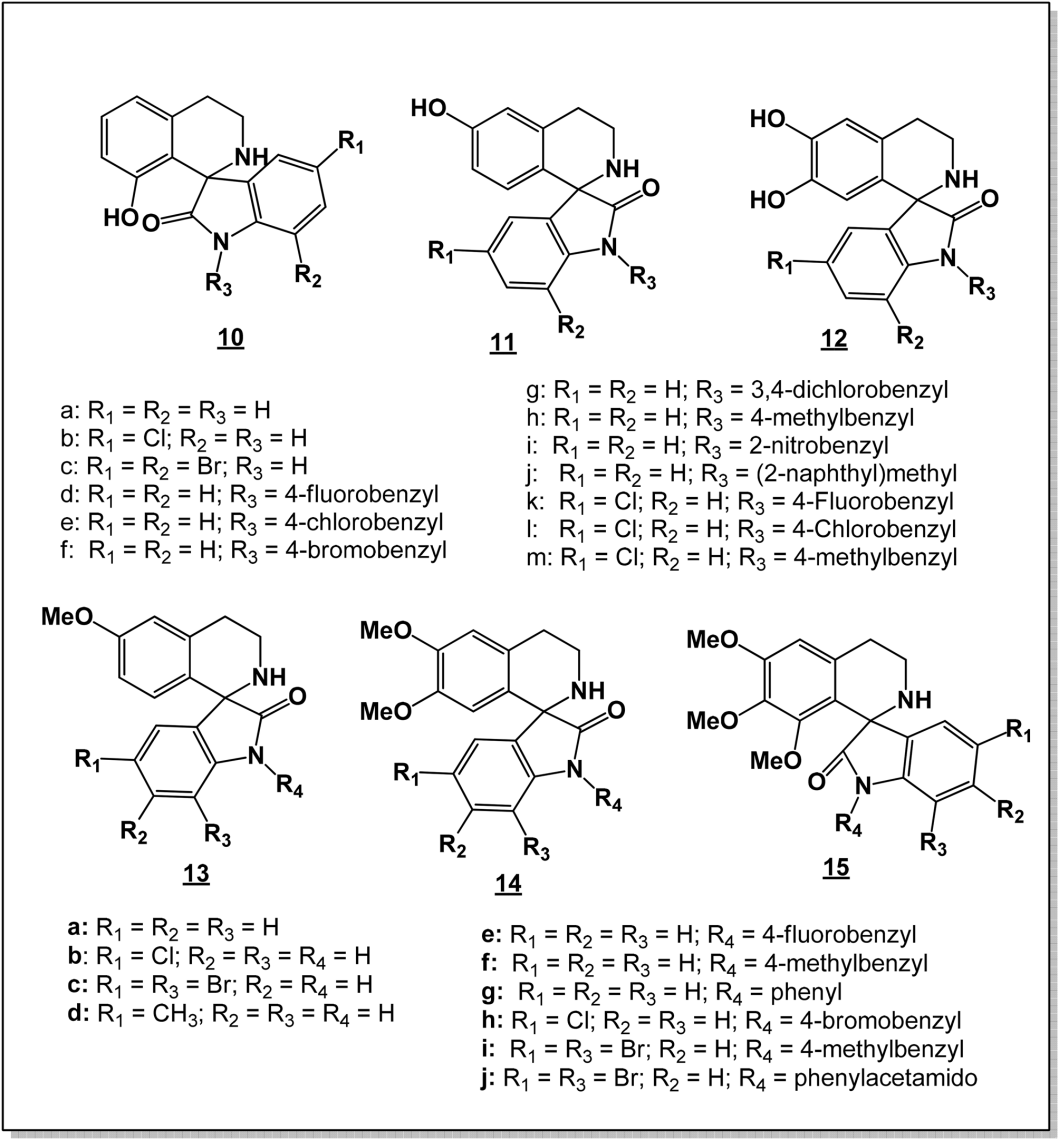
Tetrahydroisoquinoline-oxindole hybrids tested for antiplasmodial activity. *Reproduced, with modifications, from Lobe & Efange, Royal Soc. Open Sci. R. Soc. open sci. 7: 191,316. *https://dx.doi.org/10.1098/rsos.191316*; under Creative Commons Attribution License *https://creativecommons.org/licenses/by/4.0/*.*

The synthesis of the test compounds (Fig. 2) has been described^18^. The synthesis of compounds 14g and 14j is described in the Supplementary Material.

### *Plasmodium falciparum* culture and growth inhibition assay

*Plasmodium falciparum* Dd2 (multidrug resistant), NF54 (chloroquine-sensitive) and 3D7 (chloroquine-sensitive/) strains were maintained at 37 °C in 5% CO_2_ using a modified Trager and Jensen^19^ method in fresh O+ human red blood cells at 3% hematocrit in RPMI culture media containing glutamax and NaHCO_3_ (Gibco, UK), supplemented with 25 mM HEPES (Gibco, UK), 1X hypoxanthine (Gibco, USA), 20 µg/mL gentamicin (Gibco, China), and 0.5% Albumax II (Gibco, USA). When needed, parasites were synchronized at the ring stage by sorbitol (5%) treatment and further cultivated through one complete cycle (48 h) prior to drug activity studies. Compounds in dimethyl sulfoxide (DMSO) were diluted in RPMI 1640 and mixed with parasite cultures (1% parasitemia and 1.5% hematocrit) in 96-well plates to a final drug concentration of 10 μM for primary screening assays, or 10–0.078 μM for the dose–response assays. The final DMSO concentration per 100 μL culture per well was 0.1%. Artemisinin at 1 µM was used as positive drug control, while the solvent treated culture (0.1% DMSO) was used as negative drug control. Following 72 h incubation at 37 °C, parasite growth was assessed by a SYBR green I-based DNA quantification assay^20^. Briefly, 80 µL of parasitized erythrocytes were transferred to a dark plate and 40 µL of SYBR green lysis buffer (3X) added to the plate. The plate was incubated in the dark for 30 min and fluorescence was measured using a Fluoroskan Ascent multi-well plate reader with excitation and emission wavelengths at 485 and 538 nm, respectively. Mean half-maximal effective concentrations (EC_50_ values) were derived by plotting percent growth inhibition against log drug concentration and fitting the response data to a variable slope sigmoidal curve-fit function using GraphPad Prism v5.0. EC_50_ values represent means ± standard error from 2 independent assays. The inclusion of chloroquine, artemisinin or artesunate in the dose–response analyses served as experimental control.

### Cytotoxicity analyses

Cytotoxicity of the compounds was carried out using the MTT viability assay method. Normal African Green Monkey Kidney Epithelial (Vero) and the Human Liposarcoma (SW872) cells were maintained in MEM supplemented with 10% FBS, 1% Glutamine and 1% penicillin–streptomycin. Confluent cells were trypsinized and seeded at a density of 2500 cells/well (total volume 90 μL) in 96-well plates and incubated for 24 h prior to drug treatment. Two-fold serial dilutions of the compounds (100–0.0488 μM) were added to the plates and incubated under humidified conditions at 37 °C for 48 h. Viability of the cells was assessed by MTT (3-(4,5-dimethylthiazol-2-yl)-2,5-Diphenyltetrazolium Bromide) cell proliferation assay (Vybrant MTT Cell Proliferation Assay Kit V-1315) according to the manufacturer’s instructions. Absorbance of the formed formazan product was measured at 550 nm wavelength using a SUNRISE microtiter plate reader. Dose–response curves were plotted using GraphPad Prism v.5 and CC_50_ values were obtained. Selectivity indices (SI = CC_50_/EC_50_) were calculated as an indication of toxicity relative to the observed antiplasmodial activity.

### Determination of cellular mode of action

The inhibitory effects of selected compounds and reference drugs on parasite development and merozoite egress were determined by quantitative light microscopy as described^21,22^. Briefly, synchronized cultures (1.5% hematocrit and 3% parasitaemia) at different time-points (8, 30 and 42 h post invasion) were treated with either test compound or controls at 10 µM final concentration for 24 h under regular culture conditions.

Following treatment, Giemsa-stained thin smears were prepared and parasites were counted in 1,000 erythrocytes per treatment. Stage accumulation indices (stage proportion in test wells relative to same stage proportion in solvent control wells) were calculated and used to assess the in vitro effects of each compound on trophozoite and schizont development as well as merozoite egress.

### Drug effect on hemoglobin degradation

To investigate drug effects on hemoglobin degradation, early rings (6–8 hpi) or mid-trophozoite (20–24 hpi) stage *P. falciparum* parasites (1.5% hematocrit, 5% parasitemia) were co-cultured with the test compound or experimental controls (0.1% DMSO, chloroquine, artemisinin and E64) at a final concentration of 10 µM. The cultures were incubated at 37 °C and 5% CO_2_ for 24 h after which the inhibitors were removed by centrifugation at 1800 rpm for 5 min. The pellets were washed in an equal volume of 1X PBS and re-suspended in same volume of 0.1% saponin (in 1X PBS) for 3 min. Isolated parasites were pelleted at 2500 rpm for 5 min and washed twice in an equal volume of 1X PBS. The resulting parasite pellets were lysed by treatment in 20 µL of 1% Triton X-100 (in 1X PBS) for 5 min. The hemoglobin content was measured at 550 nm using a nanodrop spectrophotometer. Increases in intra-parasite hemoglobin content were then calculated relative to the solvent-treated controls.

### Drug effect on vacuolar lipid dynamics

The effect of the compounds on intracellular lipid dynamics was assessed by measurement of intracellular lipid content using Oil Red O.

Briefly, late trophozoite stage parasites (30 hpi) were treated with the compounds and controls for 24 h under regular culture conditions. Following treatment, inhibitors were removed by centrifugation at 2500 rpm for 5 min and pellets were washed using equal volumes of 1X PBS. The parasites were fixed at room temperature for 30 min with 4% paraformaldehyde and attached on poly-l-lysine coated coverslips at room temperature for 30 min. Three volumes of Oil Red O stock solution (5 mg/mL in 100% isopropanol) were diluted in two volumes of distilled water and filtered prior to use. The coverslips were then stained with diluted oil red O solution for 15 min at room temperature. Coverslips were washed thrice with 60% isopropanol and then distilled water, to remove excess dye. Thereafter, coverslips were air dried and mounted in DAPI Antifade reagent. Prepared slides were viewed under a 100× oil immersion lens and images acquired.

### Drug–drug combination analysis

Malaria parasites were continuously cultured and synchronized as earlier described prior to the assay. Drug interaction studies were performed as described^23^. Briefly, two-fold drug dilutions were prepared using a variable potency ratio drug combination approach, starting at 5EC_50_A: 0EC_50_B to 0EC_50_A: 5EC_50_B in serum-free medium, where A and B represent the different partner molecules. The ring-stage parasitized erythrocytes (∼ 10 hpi) were diluted in complete medium to 1% parasitaemia and 1.5% hematocrit and 90 μL was added in duplicate to 10 μL of the drug dilution in a 96-well plate. The test was run for 72 h and terminated when the untreated parasites were at the early trophozoite stage of the second cycle. Parasite viability was assessed by the SYBR-Green I fluorescence-based assay as earlier described. Dose–response curves and EC_50_ values of each combination and drug alone were obtained using GraphPad Prism v.5.0 and Microsoft Excel was used to calculate mean EC_50_ values and the standard error of the mean.

The obtained EC_50_ values were used to calculate 50% fractional inhibitory concentrations (FIC_50_) and the combination indices (CI) were computed from the obtained FICs.

## Results

### Discovery of (±)-moxiquindole as an antiplasmodial compound

**Figure 3:**
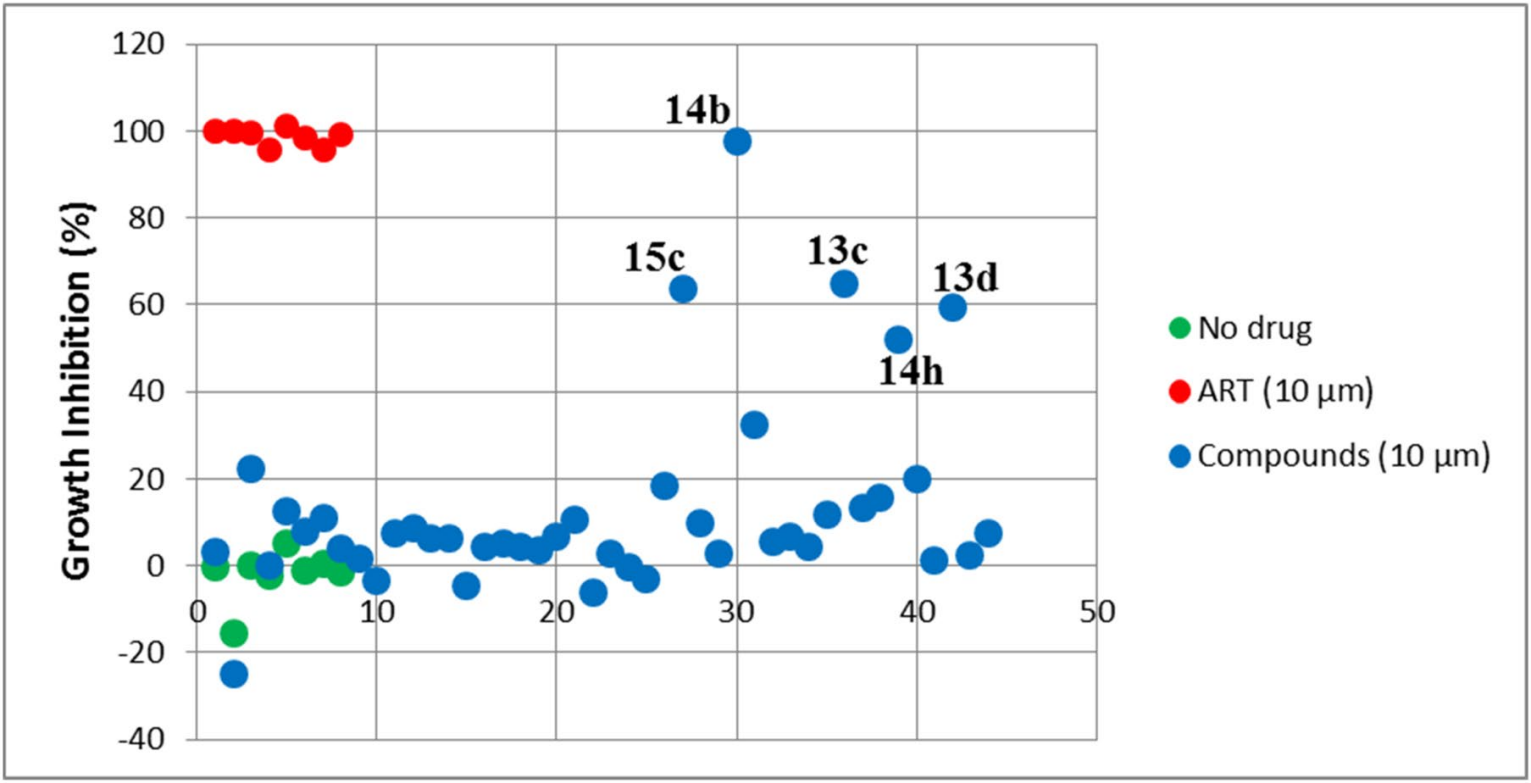
Identification of (±)-moxiquindole (14b) as most active Spiroindolone-Naphthylisoquinoline Hybrid. Dotplot indicating the ranks of each spiroindolone-naphthylisoquinoline hybrids tested in primary screens. The position of each assay well in the series (no drug, ART reference or test compound) is indicated on the x-axis.

In an effort to discover antimalarial compounds that act on new cellular targets, a library of 44 hybrid compounds incorporating structural features of two well-known antimalarial scaffolds (THIQ and oxindole) was tested against the CQ-sensitive 3D7 strain of *P. falciparum*. The SYBR green I-based DNA quantification assay was used to assess antiplasmodial activities and primary screening hits were defined as those inhibiting parasite growth by at least 50% when compared to the DMSO solvent control. Based on this criterion, 5 hits were identified (Fig. 3) and were subjected to dose–response studies on the same parasite strain. Compounds exhibiting complete dose–response curves with EC_50_ values less than 2 μM were screened against the multidrug resistant Dd2 parasite strain as well as the chloroquine-sensitive strain NF54. Four of the primary hits (13c, 13d, 14h and 15c) did not produce complete dose–response curves within the 0.078 µM to 10 µM concentration range tested, and as such were considered inactive. One hit (14b, Fig. 3), subsequently referred to as (±)-moxiquindole, emerged as the most potent with EC_50_ values of 0.686 µM, 1.865 µM and 1.730 µM against *Pf*NF54, *Pf*3D7 and *Pf*Dd2 strains, respectively (Table 2).

Cytotoxicity testing of this compound against both Vero and SW872 cell lines, showed no evidence of toxicity as selectivity indices were greater than 50-fold (Table 1), indicating in vitro safety against the tested cell lines.

**Table 1:** Antimalarial and cytotoxicity profiles of (±)-moxiquindole (MQN).

	^a^EC_50_ (µM)				^b^CC_50_ (µM)			
Compound	*PfNF54*		*Pf3D7*	*PfDd2*	*Vero*	*SW872*	^c^RI	^d^SI
CQ	0.009		0.020 ± 0.001	0.230 ± 0.028	nd	nd	11.500	nd
ART	nd		0.015 ± 0.001	0.025 ± 0.070	nd	nd	1.667	nd
AS	0.004		Nd	nd	nd	nd	nd	nd
MQN	0.686 ± 0.061		1.865 ± 0.001	1.730 ± 0.070	> 100	> 100	0.928	> 50

^a,b^Means ± SD of two independent experiments each done in duplicate.

^c^EC_50_PfDd2/EC_50_Pf3D7.

^d^CC_50_Vero/EC_50_Pf3D7.

CQ: Chloroquine, ART: Artemisinin, AS: Artesunate, MQN: (±)-moxiquindole nd: not determined RI: Resistance index, SI: Selectivity index.

### Fast-acting multistage activity of (±)-moxiquindole

The life cycle stage specific action of (±)-moxiquindole was determined by treatment of parasites at different time points followed by microscopy analysis of Giemsa-stained thin blood smears. Stage accumulation indices were calculated for each asexual parasite stage in the drug treated wells relative to the same stage in the negative control wells.

**Figure 4:**
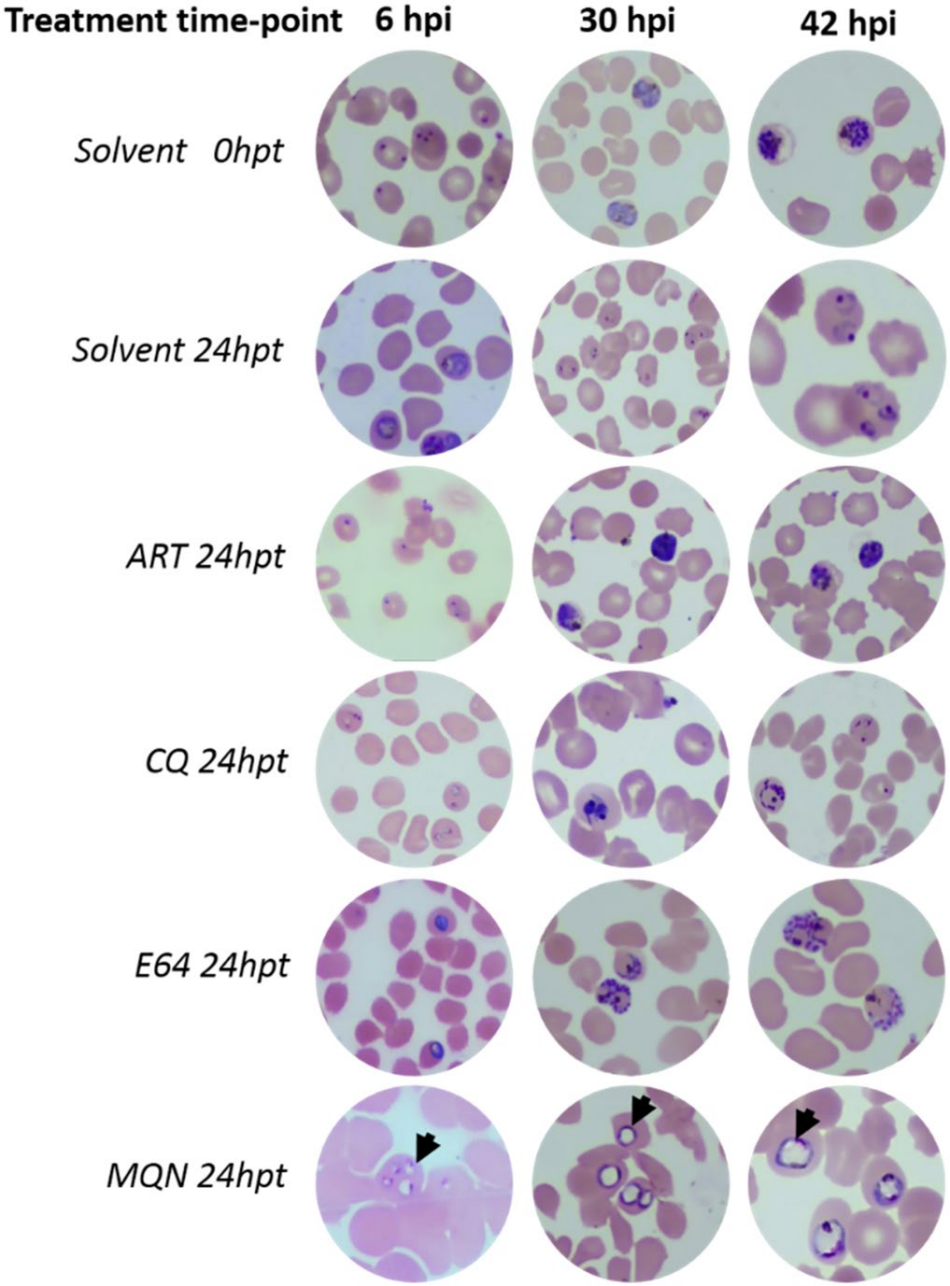
Representative images of Giemsa-stained thin smears showing drug effects on parasite life cycle progression when added at ring (6 hpi), trophozoite (30 hpi) or schizont (42 hpi). 0 hpt: treatment time point, 24 hpt: 24 h post-treatment. Arrow heads indicate unusual vacuolar structures in (±)-moxiquindole treated parasites.

The results indicate that the test compound, like artemisinin and chloroquine, exhibits fast-acting inhibitory activities against ring and trophozoite development as revealed by more than five-fold ring and trophozoite accumulation indices (Table 2). Treatment of parasites at the 42 hpi time-point resulted in a schizont accumulation index of 6.21, suggesting that (±)-moxiquindole, like the cysteine protease inhibitor E64 (l-trans-epoxysuccinyl-leucylamido-(4-guanidino), rapidly inhibits merozoite egress from the infected erythrocytes leading to accumulation of schizonts in culture. Similarly, artemisinin inhibited schizont rupture, while chloroquine had no effect, as confirmed by the appearance of ring stage parasites in the chloroquine treated cultures 24 h post-treatment at the late schizont stage (Fig. 4). Furthermore, unlike the chloroquine or artemisinin treated cultures, parasites exposed to (±)-moxiquindole showed characteristic morphological features of dying cells such as abnormal vacuolation (Fig. 4). Together, these findings suggest a novel mode of action for (±)-moxiquindole including the rapid induction of parasite vacuolation at all developmental stages tested.

**Table 2:** Stage-specific activities of (±)-moxiquindole determined by a Giemsa-based cytological profiling method.

6 hpi	R	T	30 hpi	R	T	S	42 hpi	R	S
ART	5.85	0.11	ART	0.18	7.64	0.19	ART	0.23	5.71
CQ	5.61	0.15	CQ	0.22	7.09	0.33	CQ	0.87	1.79
E64	1.12	0.95	E64	0.15	1.27	3.62	E64	0.09	6.57
MQN	5.48	0.14	MQN	0.12	7.73	0.33	MQN	0.15	6.21

Tightly synchronized *P. falciparum* parasites (3D7 strain) were treated with 10 μM final concentration of each compound at either the 6 hpi (hour post invasion, early ring), 30 hpi (mid trophozoites), or 42 hpi (midschizont) for 24 h. Giemsa-stained thin smears were prepared, parasitemia determined and stage accumulation indices for each treatment time-point was calculated. R: rings; T: trophozoites; S: schizonts.

### (±)-Moxiquindole inhibits hemoglobin degradation in *P. falciparum*

**Figure 5:**
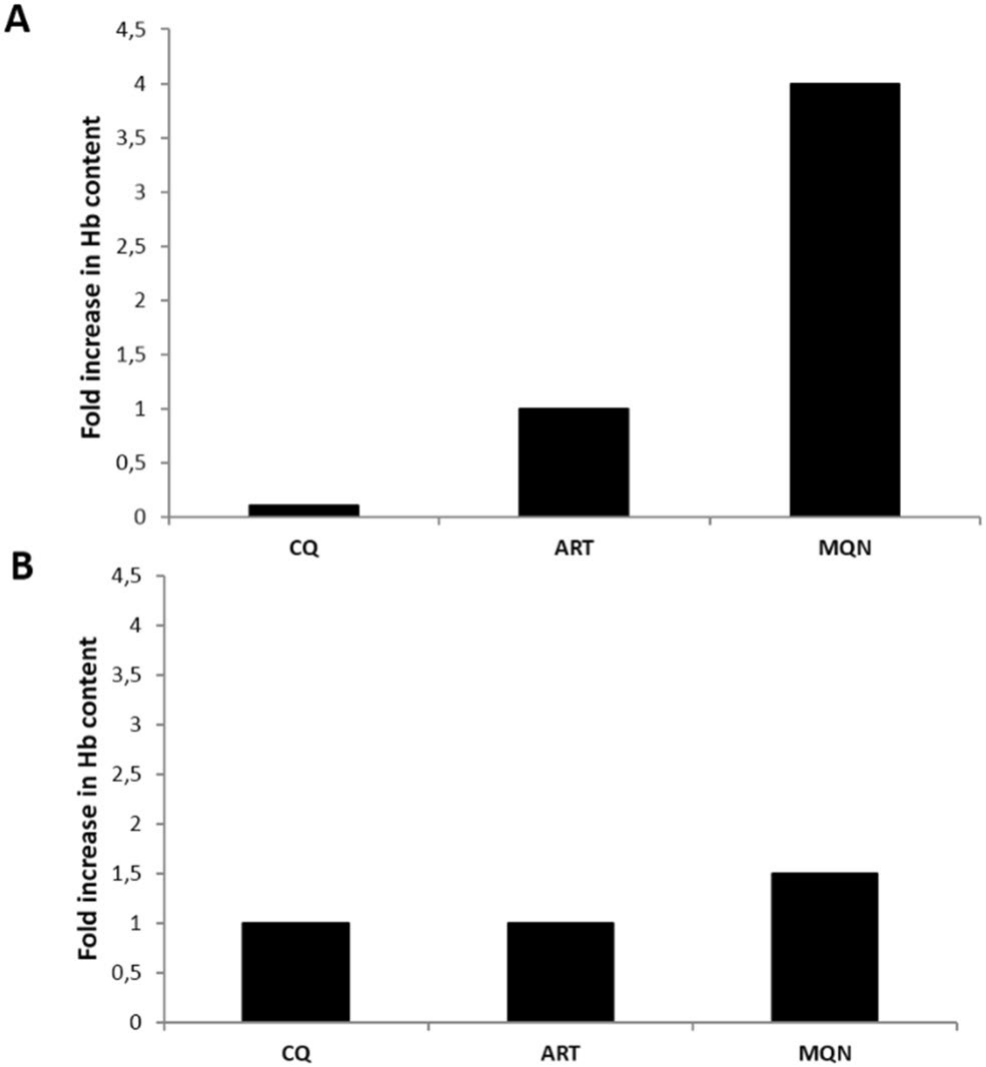
Drug effect on hemoglobin uptake and hydrolysis. *P. falciparum* rings (**A**) or trophozoites (**B**) were treated for 24 h and the hemoglobin content of saponin-isolated parasites determined by spectrophotometry. Data show fold changes in hemoglobin content of drug-treated parasites relative to E-64 treated parasites.

To determine the effect of (±)-moxiquindole on hemoglobin uptake and break down in *P. falciparum*, tightly synchronized ring or trophozoite stage parasites were treated with test compounds for 24 h followed by parasite isolation and quantification of the intraparasitic hemoglobin contents. As shown in Fig. 5, the hemoglobin content in (±)-moxiquindole-treated parasites was fourfold higher than the quantity in E-64 treated cultures when the test compound was added at the early ring stage, and 1.5-fold higher when added at the mid-trophozoite stage. Whereas artemisinin showed no detectable effect on hemoglobin accumulation in ring stage parasites, both artemisinin and chloroquine induced hemoglobin accumulation to the same degree as E-64 in trophozoite parasites. Together, these findings suggest that (±)-moxiquindole is a more effective accumulator of intra-parasitic hemoglobin in *P. falciparum*, an activity that might occur by inhibition of hemoglobin uptake and hydrolysis in the parasite’s food vacuole.

### (±)-Moxiquindole disrupts vacuolar lipid dynamics

**Figure 6:**
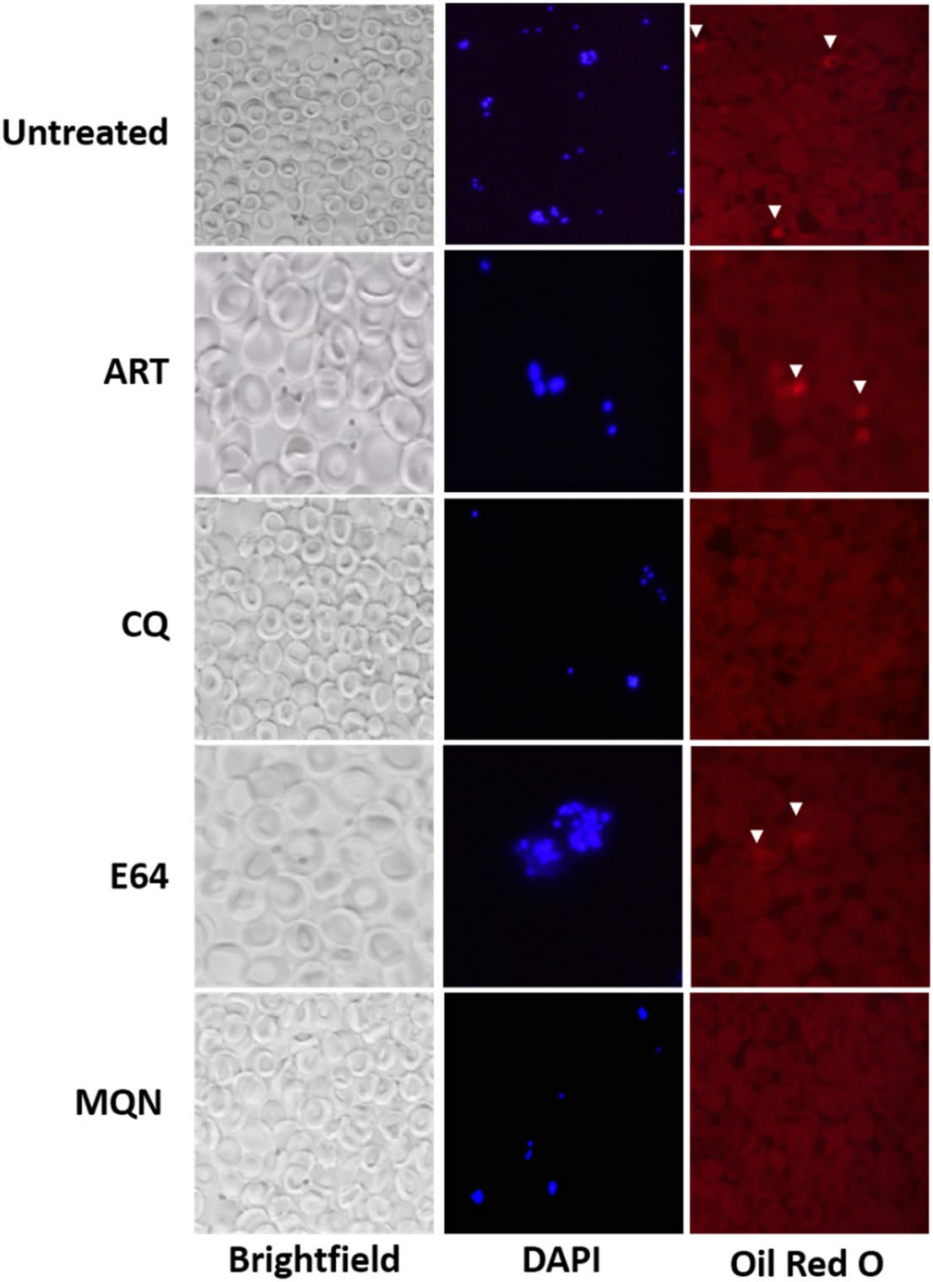
Unusual effect of (±)-moxiquindole and chloroquine on vacuolar lipid dynamics. Parasites were treated at the late-trophozoite stage (30 hpi) for 24 h and the intracellular lipid deposits were stained with Oil red O. Arrow heads indicate intracellular sites with intense staining by Oil red O. No intense staining was observed in chloroquine and (±)-moxiquindole treated cultures, suggesting an effect on vacuolar lipid dynamics.

Because cellular vacuolation may result from perturbation of intracellular lipid content, we investigated the effect of (±)-moxiquindole on vacuolar lipid dynamics using fluorescent Oil red O as lipid stain. As shown in Fig. 6, Oil red O distinctly stained intracellular spots in trophozoite and schizont stage parasites in untreated cultures as well as in artemisinin and E-64 treated cultures, but not in chloroquine and (±)-moxiquindole treated parasites. Together, these findings suggest that both chloroquine and (±)-moxiquindole may exhibit antiplasmodial activity by disrupting lipid deposition at intracellular sites proximal to the parasite digestive vacuole.

### (±)-Moxiquindole interferes with chloroquine and artemisinin actions in vitro

**Figure 7:**
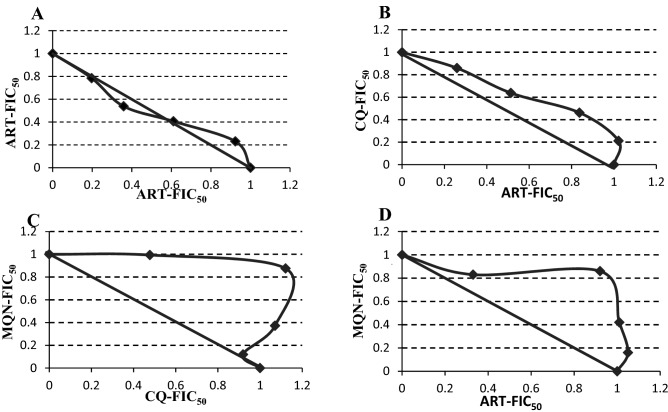
Isobolograms of the in vitro interactions between moxiquindole with chloroquine or artemisinin at fixed ratio. (**A**) Control ART/ART; (**B**) Control CQ/ART; (**C**) MQN/CQ; (**D**) MQN/ART. CQ: Chloroquine; ART: Artemisinin; MQN: (±)-Moxiquindole. FIC_50_ = Fractional Inhibitory Concentration 50. A concave curve represents a synergistic interaction, a convex is consistent with an antagonistic interaction and a straight line is consistent with an additive interaction. Axes are EC50s normalized to 1.

Given the overlapping modes of action between (±)-moxiquindole and some known antimalarial compounds in terms of speed and targeted cellular processes, we investigated the interaction between these compounds at variable combination ratios. As presented in Table 3 (cf isobolograms in Fig. 7), (±)-moxiquindole exhibited antagonistic interactions with both chloroquine (CI: 1.478) and artemisinin (CI: 1.440), similar to the antagonism observed between chloroquine and artemisinin (CI: 1.183). These results suggest possible interaction between (±)-moxiquindole and both chloroquine and artemisinin in terms of inhibited cellular processes or intracellular targets.

**Table 3:** Antagonistic interactions between (±)-moxiquindole and representative antimalarial compounds.

Drug combination	Mean EC_50_ (µM) alone	Mean EC_50_ in combination (µM)	^a^FIC_50_ A	^b^FIC_50_ B	^c^CI	Interaction
ART/ART	0.024/0.024	0.013/0.012	0.542	0.500	1.042	Additivity
ART/CQ	0.018/0.031	0.012/0.016	0.667	0.516	1.183	Antagonism
MQN/CQ	1.657/0.031	1.486/0.018	0.897	0.581	1.478	Antagonism
MQN/ART	1.657/0.018	1.374/0.011	0.829	0.611	1.440	Antagonism

^a,b^Calculated as Ʃ(mean EC_50_ of drug in combination/mean EC_50_ of drug alone).

^c^Calculated as FIC_50_A + FIC_50_B.

Additivity: CI ~ 1, antagonism: CI > 1.1, synergism: CI < 0.90.

## Discussion

The emergence and eventual spread of resistance to artemisinin-based monotherapy led to the adoption of artemisinin-based combination therapy (ACT) as the frontline treatment strategy for malaria. Unfortunately, there are increasing and worrying reports of delayed parasite clearance following the administration of artemisinin in combination with partner drugs, suggesting a slow but inexorable emergence of resistance. To address this global problem, new antimalarial drug candidates with novel mechanisms of action have to be identified and developed. The hybridization of privileged scaffolds offers a potentially useful strategy for the discovery of compounds with novel mechanisms of action. A useful example in this regard is the 2′,3′,4′,9′-tetrahydrospiro[indoline-3,1′-pyrido[3,4-b]indol]-2-one scaffold, constructed from two privileged scaffolds, 1,2,3,4-tetrahydro-β-carboline (THβC) and oxindole (OX), which has given rise to the spiroindolones, exemplified by NITD609 (KAE609, cipargarmin). Compounds of this group constitute a novel class of antimalarials which act by inhibition of *Pf* Na^+^ ATPase, a mode of action that is hitherto unknown in any other oxindole or THβC. Borrowing from this experience, our attention was drawn to the recently described 3′,4′-dihydro-2′H-spiro[indoline-3,1′-isoquinolin]-2-ones (DSIIQs) which are also constructed from two privileged scaffolds: oxindole (OX) and 1,2,3,4-tetrahydroisoquinoline (THIQ), and are therefore structurally similar to the spiroindolones and the THIQs such as the naphthylisoquinolines (NIQs). Screening of a small library of DSIIQs against CQ-sensitive *Pf* NF54 and *Pf* 3D7 strains, and multidrug resistant *Pf* Dd2 parasites resulted in the identification of a hit. The compound, subsequently referred to as (±)-moxiquindole, displayed potent inhibitory activity against all selected strains (EC_50_ *Pf* NF54 = 0.686 μM; EC_50_ *Pf* 3D7 = 1.87 µM; EC_50_ for *Pf* Dd2 = 1.73 µM), suggesting no cross-resistance with known antimalarials including pyrimethamine and chloroquine, and prompting further investigation of this compound as a representative of the DSIIQ structural class.

As a counter screen, the cytotoxicity of this compound on Normal African Green Monkey Kidney Epithelial (Vero) and the Human Liposarcoma (SW872) cell lines was evaluated using an MTT cell proliferation assay. The CC_50_ value of (±)-moxiquindole against both cell lines was > 100 μM, resulting in over 50-fold selectivity (Table 1). Subsequently, the developmental stage specific action of (±)-moxiquindole was studied by light microscopy. Following the invasion of erythrocytes, malaria parasite merozoites attain maturity by passing through a series of developmental stages termed ring, trophozoite, schizont, and segmenter. Antimalarials exert their actions by interfering with one or more stages in the life cycle of the parasite; therefore, knowledge of the stage specific action of a test compound can provide useful insights into the mode of action. In the current investigation, the mode of action of (±)-moxiquindole was compared with two antimalarials in use (ART and CQ) and E64 (a cysteine protease inhibitor), by determining their inhibitory effects on the intraerythrocytic stages of the parasite, schizont rupture and merozoite invasion. In a previous study of all antimalarials then in use, only artemisinin displayed significant inhibition of schizont maturation but no compound inhibited merozoite invasion^24^. Consistent with these reports, the present study found that artemisinin has a significant inhibitory effect on schizont maturation and eventual merozoite egress, as the schizonts appeared dead compared to the viable rings in the untreated controls. Chloroquine on the other hand, had no effect on schizont rupture as revealed by the presence of rings 24 h following drug treatment. The test compound rapidly arrested parasite development at the early ring, mid trophozoite, and schizont stages (Fig. 4). The presence of schizonts following a 24-h treatment with (±)-moxiquindole contrasts sharply with the presence of rings in the untreated controls and is indicative of an arrest in schizont rupture. Merozoite egress, which is preceded by schizont rupture, is a protease-dependent sequence of membrane permeabilization and eventual breakage^25,26^; therefore it is reasonable to attribute the blockade of schizont rupture by (±)-moxiquindole to protease inhibition. Consistent with this suggestion, the cysteine protease inhibitor E64 was also found to arrest merozoite egress in the present study (Fig. 4) as expected from previous reports^21,27^. Proteases have been implicated in a wide range of malaria parasite metabolic processes including hemoglobin degradation; therefore, additional investigations were carried out in order to gain a better understanding of the actions of the test compound. Indeed, the antimalarial activity of quinoline-based antimalarials is attributed to their interference with hemoglobin metabolism either by inhibiting the uptake of hemoglobin by the parasite, or by inhibiting degradation of hemoglobin, or by binding the toxic hematin and preventing hemozoin formation, all of which result in the eventual death of the parasite^28–30^. In the present study, both ring and trophozoite stage parasites treated with (±)-moxiquindole were found to accumulate hemoglobin to a greater extent than parasites treated with either chloroquine or artemisinin (Fig. 5). Although hemoglobin degradation is seen during schizont development, a vast majority of degradation occurs during trophozoite development. As expected, hemoglobin accumulation was higher in drug-treated ring stage parasites compared to drug-treated trophozoite stage parasites (Fig. 5). In addition, reports have shown that hemoglobin degradation is an ordered process initiated by aspartic-protease cleavages, followed by cysteine protease action. As such, the effect of cysteine protease inhibitors is minimal compared to the effect of aspartic protease inhibitors as far as hemoglobin degradation is concerned^31^. Consistent with these reports, E64; a known cysteine protease inhibitor demonstrated a minimal effect on hemoglobin degradation compared to the test compound. These observations further support the view that (±)-moxiquindole is indeed acting as a protease inhibitor and a more effective accumulator of intra-parasitic hemoglobin; thus, depriving the organism of an essential source of substrates for energy metabolism.

Further support for this proposed mode of action is provided by the combination studies which paired (±)-moxiquindole with either chloroquine or artemisinin. As presented above, the interaction of the test compound with either antimalarial was antagonistic (Table 3). The actions of both chloroquine and artemisinin depend on the presence of heme, a metabolite generated by protease catalyzed degradation of hemoglobin. Drug induced blockade of the latter process, with resulting depletion of heme levels, would therefore be expected to negatively affect the actions of these two compounds as observed in the present study. Similar observations have been reported in a study of the interaction between chloroquine and both aspartic and cysteine proteases using the same analytic methods^32^.

Previous studies have showed that during intraerythrocytic development, the phospholipid content of the parasite increases significantly as the latter accumulates membranes needed for growth and division^33^. Though some of the lipids are synthesized in the apicoplast, the bulk of the lipids are scavenged from the host. These lipids are metabolised to generate neutral lipid bodies which nucleate the formation of hemozoin in the parasite digestive (food) vacuole^34^. Consequently, the lipid metabolic pathway is an important target in antimalarial drug discovery. In the continuing investigation of the mode of action of (±)-moxiquindole, the actions of this compound on lipid metabolism were compared with those of chloroquine, artemisinin and E64. Trophozoite stage parasites treated with either, artemisinin or E64 clearly revealed the presence of stained neutral lipid bodies indicating that these compounds have no effect on vacuolar lipid uptake. On the other hand, parasites treated with either chloroquine or the test compound were devoid of neutral lipid bodies clearly suggesting interference with parasite vacuolar lipid dynamics (Fig. 6). This supports previous findings that chloroquine and other quinoline-based compounds bind toxic ferriprotoporphyrin IX and inhibit dimerization^35^.

In conclusion, preliminary investigation of (±)-moxiquindole, a representative compound of the recently described privileged scaffold hybrid 3′,4′-dihydro-2′H-spiro[indoline-3,1′-isoquinolin]-2-one (DSIIQ), strongly suggests that the antimalarial activity of the compound can be attributed to (1) protease inhibition, which results in the blockade of hemoglobin degradation with subsequent starvation of the parasite, and (2) interference with parasite lipid dynamics. The DSIIQs are structurally related to both the spiroindolones and the NIQs. Previous investigations have attributed the antimalarial activity of NIQs to interference with hemoglobin metabolism. Based on the results discussed above, it would therefore appear that at least some of the actions of (±)-moxiquindole are similar to those of the NIQs. Subsequent studies will compare the actions of this compound with those of the structurally related spiroindolones and concentrate on improving the antimalarial activity by modification of chemical structure. The available evidence suggests, nevertheless, that this new hybrid scaffold has the potential to yield an interesting group of antimalarial agents.

## Supplementary information

Supplementary information.
