# Supplementary material for: Spirofused tetrahydroisoquinoline-oxindole hybrids as a novel class of fast acting antimalarial agents with multiple modes of action
Source: Sci Rep. 2020 Oct 21;10:17932. doi: 10.1038/s41598-020-74824-0 (PMC7578093; doi:10.1038/s41598-020-74824-0)
Supplement: Supplementary file 1 — Supplementary information. [file 41598_2020_74824_MOESM1_ESM.docx]

**SUPPORTING INFORMATION**

**Spirofused Tetrahydroisoquinoline-Oxindole Hybrids as a Novel Class of Fast Acting Antimalarial Agents with Multiple Modes of Action**

Noella M. Efange,^1,2^ Maloba M. M. Lobe,^3^ Rodrigue Keumoe^2^, Lawrence Ayong,^2*^ and Simon M. N. Efange^3^*

^1^Department of Biochemistry & Molecular Biology, University of Buea, P.O. Box 63, Buea, Cameroon.

^2^Centre Pasteur du Cameroon, Yaoundé, Cameroon.

^3^Department of Chemistry, University of Buea, P.O. Box 63, Buea, Cameroon.

*Corresponding authors:

Lawrence Ayong, Centre Pasteur du Cameroun, Yaoundé, Cameroon

*Email: ayong@pasteur-yaounde.org*

Simon M.N. Efange, Department of Chemistry, University of Buea, P.O. Box 63, Buea, Cameroon.

*Email: [efange.mbua@ubuea.cm](mailto:efange.mbua@ubuea.cm)*

**Table of Contents**

**Page Number**

**Chemistry………………………………………………………………………..S1-S3**

**Synthesis of 6',7'-dimethoxy-1-phenyl-3',4'-dihydro-2'H-spiro[indoline-3,1'-isoquinolin]-2-one (14g).**

The compound was synthesized from 1-phenylisatin (0.5 g, 2.2 mmol), 3,4 - dimethoxyphenethylamine (0.5 g, 2.6 mmol) and polyphosphoric acid (2 g) as previously described (Lobe & Efange, 2020; Method G). The crude product was purified by flash chromatography (hexane : ethyl acetate—60 : 40). Yield, 0.72 g, 85% (brown solid), M.p. 227 – 229 ^o^C (HCl salt).

**^1^H NMR** (DMSO-d_6_, 600 MHz): δ ppm 1.94 (ddd, , *J* = 16.4, 5.8, 3.6 Hz, 1H, H1’ ), 2.13 (ddd, *J* = 15.8, 9.1, 5.8 Hz, 1H, H4’a), 2.31 (dt, *J* = 12.6, 4.6 Hz, 1H, H3’a), 2.62 (m, 3H, 7’-OCH_3_), 2.88 – 2.96 (m, 5H, H3’b, H4’b, 6’-OCH_3_), 5.21 (d, *J* = 2.5 Hz, 1H, H8’), 5.90 (d, *J* = 2.5 Hz, 1H, H5’), 5.95 (dd, *J* = 7.9, 2.6 Hz, 1H, H7), 6.21 (td, *J* = 7.6, 2.7 Hz, 1H, H5), 6.35 (dd, *J* = 7.6, 2.8 Hz, 1H, H4), 6.37 -6.41 (m, 1H, H4”), 6.54 – 6.59 (m, 3H, H6, H2”, H6”), 6.65 – 6.69 (m, 2H, H3”, H5”). **^13^C NMR**  (DMSO-d_6_, 150 MHz): δ ppm 26.8 (C4’), 37.6 (C3’), 54.2 (7’-OCH_3_), 54.3 (6’-OCH_3_), 62.6 (C3/C1’), 108.4 (C8’), 108.5 (C7), 111.5 (C5’), 122.9 (C5), 123.9 (C4), 124.9 (C8’a), 125.8 (C2”, C6”), 127.4 (C3a), 128.3 (C4”), 128.3 (C4’a), 128.7 (C3”, C5”), 133.1 (C6), 133.5 (C1”), 143.0 (C7a), 147.0 (C7’), 148.1 (C6’), 177.3 (C2). **FTMS + cESI:** m/z 387.17 [M+1]^+^.

**Synthesis of 2-(5,7-dibromo-6',7'-dimethoxy-2-oxo-3',4'-dihydro-2'H-spiro[indoline-3,1'-isoquinolin]-1-yl)-N-phenylacetamide (14j).**

The compound was prepared from 2-(5,7-dibromo-2,3-dioxoindolin-1-yl)-N-phenylacetamide (2 g, 4.6 mmol), 3,4 - dimethoxyphenethylamine (0.83 g, 4.6 mmol) and polyphosphoric acid (3 g), as previously described (Lobe & Efange, 2020, Method G). The crude product was purified by flash chromatography (hexane : ethyl acetate—60 : 40). Yield, 1.34 g, 49% (black solid); M.p. 202 – 203 ^o^C (HCl salt).

**^1^H NMR** (CD_3_OD, 700 MHz): δ ppm 2.84 (dt, *J* = 16.1, 4.6 Hz, 1H, H4’a), 3.02 (ddd, *J* = 16.2, 8.9, 5.3 Hz, 1H, H4’b), 3.23 (dt, *J* = 12.7, 4.8 Hz, 1H, H3’a), 3.67 – 3.74 (m, 4H, H3’b, 7’-OCH_3_), 3.82 (s, 3H, 6’-OCH_3_),

4.97 (m, 1H, CH_2_-Ar ), 5.02 (m, 1H, CH_2_-Ar ), 6.54 (s, 1H, H8’), 6.78 (s, 1H, H5’), 7.10 (m, 1H, H4”), 7.29 – 7.33 (m, 3H, H4, H3”, H5”), 7.54 – 7.56 (m, 2H, H2”, H6”), 7.67(d, *J* = 1.9 Hz, 1H, H6). **^13^C NMR**  (CD_3_OD, 175 MHz): δ ppm 27.5 (C4’), 38.6 (C3’), 44.1 (CH_2_-C=O ), 55.0 (7’-OCH_3_), 55.2 (6’-OCH_3_), 63.1 (C3/C1’), 103.1 (C7), 109.7 (C8’), 111.9 (C5’), 115.7 (C5), 119.6 (C2”, C6”), 123.8 (C4”), 124.8 (C8’a), 127.0 (C4), 128.4 (C4’a), 128.5 (C3”, C5”), 136.1 (C6), 138.3 (C1”), 139.1 (C3a), 139.9 (C7a), 148.1 (C7’), 148.8 (C6’), 166.1 (CH_2_-C=O), 179.3 (C2). **FTMS + cESI:** m/z 602.01 [M+1]^+^.
